# High school students' STEM interests and career as*p*irations in Qatar: An exploratory study

**DOI:** 10.1016/j.heliyon.2023.e13898

**Published:** 2023-02-23

**Authors:** Abdellatif Sellami, Malavika Santhosh, Jolly Bhadra, Zubair Ahmad

**Affiliations:** a Educational Research Center, College of Education, Qatar University, P.O.Box 2713, Doha, Qatar; b Qatar University Young Scientists Center (QUYSC), Qatar University, P.O.Box 2713, Doha, Qatar

**Keywords:** STEM subjects, Career aspirations, Quantitative analysis, High school students, Qatar

## Abstract

This study sought to explore high school students' interest in science, technology, engineering, and mathematics (STEM) related disciplines and careers in the context of Qatar. Quantitative data was collected using a survey of 1492 high school students in grades 11–12. The normality tests (Shapiro-Wilk test and Kolmogorov Smirnov test) revealed the non-normal distribution of data, leading to employing non-parametric analyses, including Mann Whitney *U* test, Kruskal Wallis H, and logistic regression. Results indicated that whereas students' interest in mathematics and science subjects was aligned with their likelihood to pursue STEM careers, however, their interest in engineering and technology doesn't line up with their STEM career aspirations. The findings also revealed the variability of students' STEM interests across gender and nationality. In general, female students exhibited higher STEM interests than their male counterparts, while specially expatriates were more inclined toward STEM than Qatari nationals. Overall, these findings postulate the need to improve the exposure of males in general and Qatari nationals specifically to STEM fields of study, particularly the subjects of engineering and technology, to meet the goals of Qatar's National Vision 2030.

## 1 Introduction

The myriad challenges being faced by our rapidly changing world have heightened the demand for creative and innovative solutions. To solve many complex real-world ills, there is a pressing need for professionals who possess critical 21st-century skills and competencies. These skills are highly required to address existing and possible global problems to do with energy, the climate, the environment, and health, to name a few. More than ever before, the fields of science, technology, engineering, and mathematics (STEM) have taken center stage in educational and policy circles as potential instruments that may aid in solving these issues [1].

However, the scarcity of STEM professionals being trained globally poses a significant concern requiring global attention [1]. In recent years, there has been a reportedly considerable decrease in the number of students interested in STEM careers [2,3]. There is no universal definition for STEM careers. However, researchers believe that STEM workers use their knowledge of science, technology, engineering, or mathematics to try to understand how the world works and solve problems [4]. Many countries are already facing problems attracting individuals to STEM-based career pathways [5]. Correspondingly, schools and teachers worldwide are struggling to keep students interested and motivated in STEM as a field and career pathway [6].

Similar concern among Gulf countries regarding the shortage of qualified STEM workers has been raised [7]. Presently, these countries have been focused on efforts to diversify their economy to reduce their dependence on fossil fuels in favor of more sustainable sources. Economic diversification is necessary for these countries to transform into knowledge-based economies [8]. As the knowledge-based economy model is profoundly dependent on the STEM industry, there is a great need for STEM skills among citizens. Although educational reforms in the field of STEM education are emerging in Qatar [9,10], the consequences are still not as anticipated. According to the Ministry of Development Planning and Statistics in Qatar, the number of national students graduating from non-STEM programs (i.e., accounting, economics, and banking) at public colleges and universities in Qatar is greater than the number of students graduating with STEM degrees (i.e., chemistry, biology, and biomedical sciences) [11]. In this context, it is also noteworthy that the country's school curricula don't incorporate engineering and technology subjects, thereby don't adequately train students in the same [11]. Followingly the nationals' preferences for public and business-oriented careers [12,13], have made Qatar dependent on expatriates for STEM-related professions [10]. As this might affect the country's economic development, it becomes significantly critical to identify and understand the factors affecting STEM interests and career aspirations among the young generations.

Therefore, Qatar's concerns over the fewer nationals in STEM majors and the STEM workforce have motivated us to conduct this STEM-interest-career-based quantitative study [14]. Which in turn, would help us understand students' level of interest in STEM for better STEM educational transformations. As high school is the most critical age for cultivating STEM career interests [15,16], the authors have designed a STEM interest and career survey to explore the STEM interests and career aspirations of high school students in Qatar. Wherein the study sought to explore if their STEM interest aligns with their STEM career aspirations. In addition, the variability of STEM interest across the variables such as students' grades, gender, nationality, school, and parents' occupation has also been examined.

### 1.1 Literature review and conceptual framework

Both STEM interests and career aspirations are influenced by personal (gender, race, nationality, parent education/occupation, etc.), environmental (school-related factors), and motivational factors (expectation, self-efficiency, etc.) [17,18]. In addition, Blonticky et al. (2018) argued that the student's STEM interest affects their STEM career aspirations [19]. Therefore, based on previous literature, our study sought to analyze six potential determinants (personal and environmental factors) of STEM interests i.e., nationality, gender, school, grade, and fathers' and mothers' occupations.

Gender is a potential factor that influences students’ STEM interests and career aspirations. Where the bulk of research evidence states that male students are more inclined toward STEM fields [20,21]. Whereas female students seem to be more interested in arts and education fields [20,21]. This gender gap is most prominent in engineering fields, favoring males [21]. Interestingly the study findings by Weibe et al., 2018, have revealed that males show greater interest in physical sciences/engineering, while females do in biological/clinical sciences [22]. Along with gender, this study investigated other relevant personal factors such as nationality and grade. Literature has shown mixed relationships between age/grade and STEM interest & career aspiration development [23,24]. Christensen et al., 2014 have shown that 11th graders are more likely to have positive STEM attitudes than 12th graders [25]. In addition, the nationality factor was opted to explore the ethnic variation in STEM and non-STEM professions by Qataris and expatriates [10,12,13]. In general, African, American, and Asian students were significantly more likely to maintain an early STEM interest relative to White students [26].

Similarly, parents are also one of the most influential factors in determining students' STEM interests and career decision-making [27,18,28]. Cridge and Cridge [2] state that parental education and occupation has a relationship with students' interest and career choices. The parental occupation in STEM is more likely related to higher science-related aspirations among their children [29]. A systematic review and narrative analysis by Plasman et al. [29], have shown a positive relationship between the parents employed in STEM occupations and the success/persistence of high school students in STEM fields. In addition, their study showcased that female and minority students with parents employed in STEM occupations are more likely to benefit than their male counterparts’ students [29]. STEM-employed parents may inspire their children toward STEM directly by sharing knowledge and career guidance and indirectly by providing support, encouragement, and family bonding [30].

Correspondingly, the school type and classroom activities (environmental factor) also tend to impact students' STEM interests and career aspirations. School environment and curriculum that actively supports formal and informal scientific investigations aid in nurturing STEM interests and career aspirations [3,18,29,31,32]. A longitudinal study by Ketenci et al. [33], has shown variability in STEM aspirations in public and private schools [33]. Their study evaluated the students’ choice of STEM aspirations with gender, math self-efficacy, socioeconomic status (SES), school type, and urbanicity as predictors. Concludingly, male students are more likely to choose a STEM-related career in a private school with high SES and math self-efficacy [33].

Most of the studies exploring the determinants of STEM interests and careers are based on the Social Cognitive Career Theory (SCCT) [27,18,34]. Therefore, the theoretical background of the study also relies on the SCCT, which was developed based on Bandura's social cognitive theory [35], introduced by Lent and other researchers [36,37]. The theory reports that students' STEM-related career aspirations depend on major constructs (motivational factors) and sub-constructs (personal and environmental factors) [36,37]. This preliminary study of Qatar sought to explore and comprehend the sub-constructs, related to SCCT. Thus, the research questions addressed in this study are as follows:

1. What careers do high school students in Qatar aspire to?
2. What are the likely influences of high school students' grade level, school type, gender, nationality, and parental occupation on their STEM interests?
3. Do the career aspirations of Qatar's high school students align with their STEM interests?

## 2 Research methods

This exploratory study uses quantitative data collected via a survey questionnaire distributed to 11th and 12th-grade students enrolled in government and private high schools in Qatar.

### 2.1 Participants

This study was carried out in Qatar's public (government) and private schools. Upon obtaining the ethics approval from Qatar University Institutional Review Board (QUIRB), official letters were sent to school board superintendents and teachers requesting permission to gather student data in their respective schools. In total, 1492 high school students in Qatar participated in the study. Non-probability uncontrolled convenient sampling was employed, due to time constraints, ease of availability, and the study is preliminary and exploratory.

Participants in the survey included 11th and 12th-grade national (Qatari) and expatriate students in public and private schools in Qatar. Table 1 demonstrates the demographic distribution of the survey participants. Looking at students' gender, 60.53% of the participants were females, and 39.47% were males. Regarding students' grade level, 48.89% were in grade 11, and 51.11% were in grade 12. Most participants were expatriate students (83.23%), compared to only 13.77% of Qatari students. The results further revealed that more than half (66.58%) of the male guardians occupied non-STEM professions and that 45.52% of the female guardians were housewives.

**Table 1:** Demographics of the respondents (N = 1492).

Variable	Missing responses	Groups	%	No.
Gender	0	Male	39.47	589
		Female	60.53	903
School	34	Government	36.21	528
		Private	63.79	930
Grade	9	11th	48.89	725
		12th	51.11	758
Nationality	6	Qataris	13.77	206
		Expatriates	83.23	1280
Father's Profession	352	STEM profession	33.42	381
		Non-STEM profession	66.58	759
Mother's Profession	895	STEM profession	23.61	141
		Non-STEM profession	60.30	360
		Housewife	16.08	96

**Note:** Students' res*p*onse to housewife means their mothers are not involved in any *p*aid STEM or non-STEM jobs.

### 2.2 Procedure

In executing the survey, three main steps were followed: (1) survey formulation, (2) survey piloting, and (3) survey implementation. The survey questions by the research team are based on the available literature [19,38]. And the STEM-career-interest survey designed by Kier et al. [34]. The survey utilized in our study consisted of 24 questions of a Likert scale format, comprising three main parts: demographics, STEM interests (15 items), and STEM career aspirations (1 item) (see Fig. S1). Questions based on STEM interests were of the 5-point Likert scale type. Likert scales ranging from −2 to +2 were coded as per the statements: 2 = “Strongly Disagree”, −1 = “Agree”, 0 = “Undecided”, 1 = “Agree”, and 2 = “Strongly Disagree”. The questions about STEM career aspirations were open-ended (see Fig. S1). For analysis purposes, students' responses were recoded as 1 = “STEM career” and 0 = “non-STEM career” aspirations. The students' responses were coded per the International Standard Classification of Occupations (ISCO-88) [4]. Similarly, for questions such as students' gender (male, female), nationality (national, expatriate), majors (STEM, non-STEM), guardian's profession (STEM, non-STEM), grade (11th, 12th), etc., binary coding (1 and 0) was used.

The second step involved piloting the survey with two focus groups (Arabic and English) to finalize the survey instrument. Discussions with the focus groups aided us in identifying and addressing issues related to the clarity and wording of questions. This eventually helped us rewrite questions to ensure the language and content of the questions. The third and final step in the implementation of the survey consisted of distributing the questionnaires after receiving student and parent-signed consent forms and formal approvals from teachers and school administrators. Students were given a choice to respond to the survey in English or Arabic. It was ensured due to different mediums of instruction in school (Arabic and English in public and private schools respectively). The average time taken to complete the survey was 15–30 min.

### 2.3 Data analysis

After collecting data, participants' responses were coded, saved, and analyzed using SPSS (the Statistical Package for The Social Sciences) software. Descriptive statistics were evaluated for the overall analysis of the data. To ascertain the reliability of our data, we used different statistical tests based on the type of data we were looking at. Firstly, a Cronbach Alpha test was performed to measure the reliability of the questions used for analysis in the study. The alpha values computed for each survey construct are given in Table 2. The reliability test revealed that all the questions used for analysis were reliable. *Alpha* values above 0.70 are regarded as reliable, and those above 0.8 are considered highly reliable [39].

**Table 2:** Instrument reliability test for STEM interest.

Survey parts	Dimension	No. of items	Cronbach's Al*p*ha	Reliability
STEM interest	Science interest	5	0.781	Reliable
	Mathematics interest	5	0.721	Reliable
	Engineering & technology interest	5	0.844	Highly reliable

Secondly, the data's normality (Kurtosis and Skewness) was evaluated using the Shapiro-Wilk and Kolmogorov-Smirnov tests (see Table 3). For the normality test, students' STEM interest score (SIS) was identified as the dependent variable developed by summing students' interest in science, mathematics, engineering, and technology. The independent variables were the school type, gender, grade, nationality, and parents' occupation. The normality tests were significant (*p* < 0.05), revealing the distribution to be not normal. Bearing in mind that our null hy*p*othesis states that the data is normally distributed, a *p*-value less than 0.05 rejects the hypothesis. It supports our claim that the data is not normally distributed, thus allowing the use of non-parametric data analysis tools.

**Table 3:** Test of normality for dependent variable SIS (STEM interest score) and independent variables (gender, nationality, grade, school, parental occupation).

Dependent variable	Independent variable	Kolmogorov-Smirnov			Shapiro-Wilk		
		Statistics	Df	Sig.	Statistics	Df	Sig.
Independent variable - School type							
STEM interest score	Government	.080	314	<.001	.971	314	<.001*
	Private	.053	518	.001	.988	518	<.001*
Independent variable – Grade							
STEM interest score	11th	.065	425	<.001	.977	425	<.001*
	12th	.063	407	<.001	.985	407	<.001*
Independent variable: Gender							
STEM interest score	Male	.069	342	<.001	.980	342	<.001*
	Female	.060	490	<.001	.983	490	<.001*
Independent variable: Nationality							
STEM interest score	National	.095	77	.003	.955	77	.009*
	Expatriate	.060	755	<.001	.983	755	<.001*
Independent variable: Father's occupation							
STEM interest score	STEM profession	.056	317	.016	.984	317	.001*
	Non-STEM profession	.075	515	<.001	.979	515	<.001*
Independent variable: Mother's occupation							
STEM interest score	STEM profession	.065	292	.004	.972	292	<.001*
	Non-STEM profession	.138	43	.038	.940	43	.022*
	Housewife	.062	395	<.001	.991	395	.016*

Note: Df = degree of freedom; * Statistically significant; Sig. = significance level at 0.05.

Thirdly, non-parametric tests were also employed in the data analysis, including the Mann-Whitney *U* test, Kruskal Wallis H [38], and the bivariate logistic regression model. The Mann Whitney U and Kruskal Wallis H tests were used to see if there exists any change in STEM interests across the groups (school, gender, grade, nationality, parent's occupation). The binary logistic regression analysis was used to find the correlation between the students' career aspirations and STEM interests.

## 3 Results

### 3.1 What careers do high school students in Qatar aspire to?

**Fig. 1:**
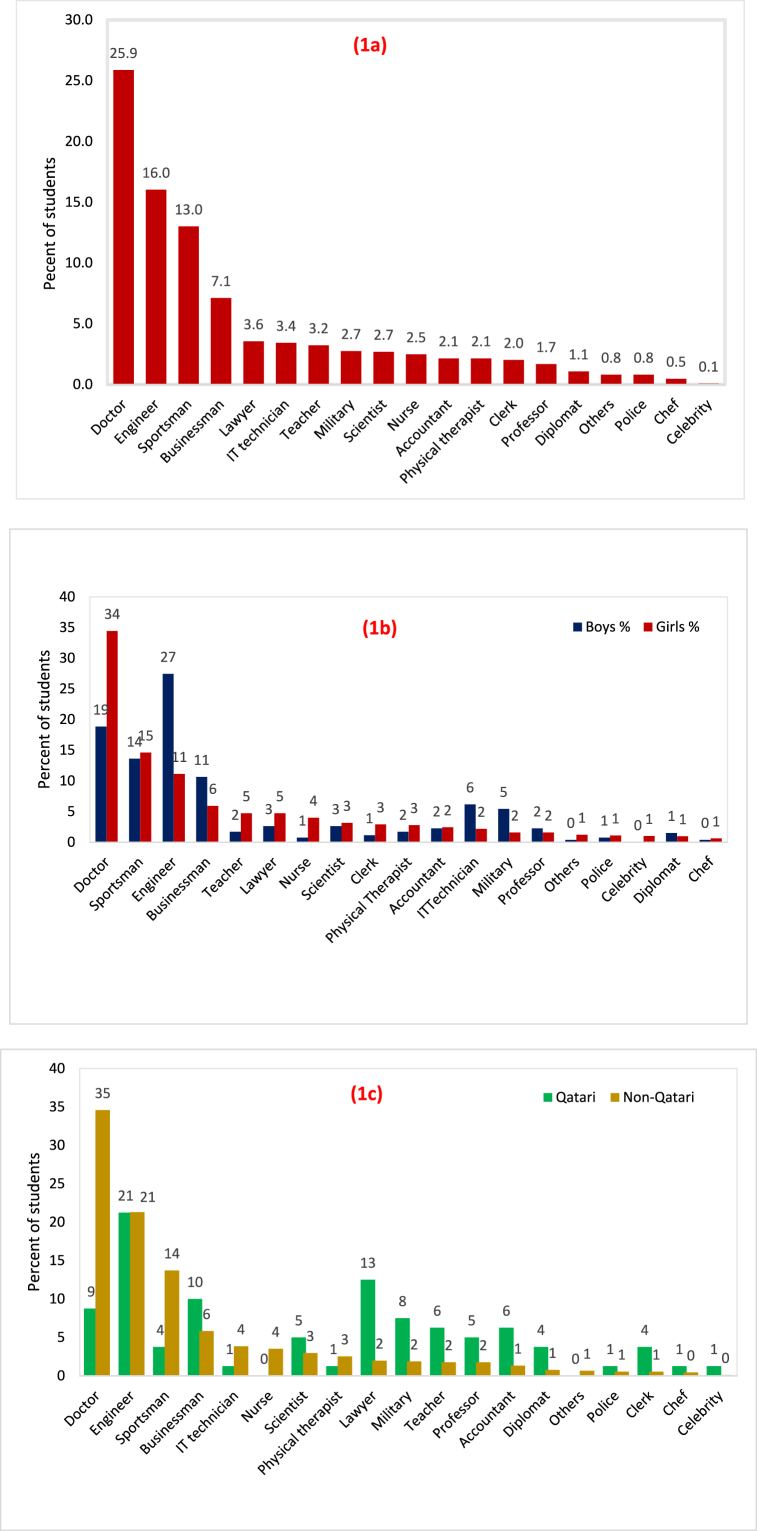
**(a)** High school students' career aspirations. **(b)** High school students' career aspirations by gender, and **(c)** High school students' career aspirations by nationality.

Fig. 1(a) demonstrates various career aspirations of high school students in Qatar. The results indicated that 26% of high school students aspire to be doctors, followed by those who reported aspirations to a career in engineering (16%) and sports (13%). Our study's results further disclosed that the career the students aspire to the least is becoming a celebrity (0.1%). Interestingly, most females aspire to be doctors and males to be engineers (Fig. 1(b)). Fig. 1(c) reveals the national differences. Most of the expatriate students aspire to be a doctor while the same percentage of both Qatari and expatriate students aspire to be engineers. The second highest careers aspirations of Qatari students are to be a lawyer.

**Fig. 2:**
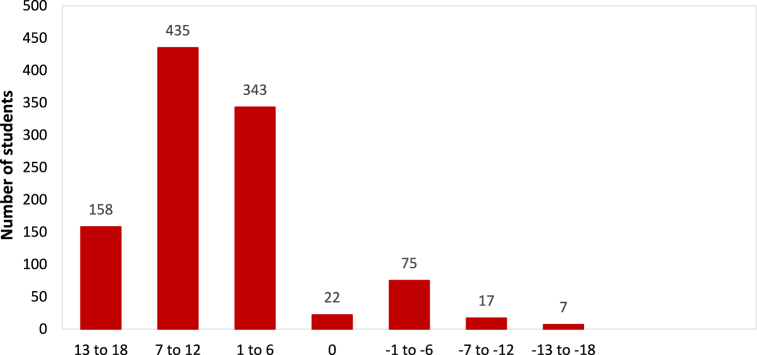
Students' STEM interest score (SIS).

Furthermore, students' responses to the question related to career aspirations were sub-categorized into STEM and non-STEM careers and were coded as 1 and 0, respectively. This was done to examine the proportion of students that aspire to STEM versus non-STEM careers. The results reveal that the percentage of high school students with STEM and non-STEM career aspirations is 55.3% and 44.7%, respectively (Fig. 2). Admittedly, while the results reveal that overall STEM career aspirations are ∼10% higher than the non-STEM, however, few students are graduating in STEM majors from universities and colleges (11). The expatriate students' STEM careers interest is higher than the non-STEM, this trend is the other way around in Qatari national students (see Table 4). The gender-based differences illustrated that the expatriate male & female students show a closely alike proportion of inclination towards STEM careers. Whereas Qatari females described higher STEM career aspirations as compared to Qatari male students.

**Table 4:** The proportion of high school students’ shoed aspirations to a career in STEM and non-STEM fields.

Variable	Sub-variable	Percentage of students (%)	
		STEM career aspirations	Non-STEM career aspirations
Qatari Nationals	Male	33.33	66.67
	Female	45.21	54.79
	Overall	37.50	62.50
Expatriate	Male	65.10	34.99
	Female	67.23	32.77
	Overall	68.72	31.28
Overall	Male	63.83	36.17
	Female	63.85	36.15
	Overall	55.30	44.70

### 3.2 What are the likely influences of high school students' grade level, school type, gender, nationality, and parental occupation on their STEM interests?

To determine if the students' STEM interests vary across their gender, grade, school type, nationality, and parental occupation, the non-parametric test, Mann-Whitney U was performed. By contrast, the Kruskal-Wallis H test was carried out in cases where more than two independent categories were examined. Table 5 illustrates the Mann-Whitney U and Kruskal-Wallis H test for the independent variables (gender, nationality, school type, grade, and parental occupation).

**Table 5:** Mann-Whitney U and Kruskal-Wallis H test, examining the STEM interest across the various independent variables.

	Variables	Grou*p*s	Test Statistics	Values
1.	Gender	Male	Mean rank	440
		Female	Mean rank	635
			Mann-Whitney U	110998.500
			*p*-value	<0.01*
2	Nationality	National	Mean rank	468.12
		Expatriate	Mean rank	544.31
			Mann-Whitney U	57049.500
			*p*-value	0.018*
3	Grade	11th	Mean rank	541
		12th	Mean rank	530.89
			Mann-Whitney U	140658.000
			*p*-value	0.592
4	School	Government	Mean rank	530.15
		*P*rivate	Mean rank	529.14
			Mann-Whitney U	128123.500
			*p*-value	0.959
5	Father's Occupation	STEM *p*rofession	Mean rank	424.25
		Non-STEM *p*rofession	Mean rank	431.86
			Mann-Whitney U	87665.000
			*p*-value	0.663
6	Mother's occupation	STEM *p*rofession	Mean rank	549.86
		Non-STEM *p*rofession	Mean rank	515.02
		Housewife	Mean rank	522.46
			Kruskal Wallis test	11.541
			*p*-value	0.173

**Note:** * Statistically significant; Sig. = significance level at 0.05.

The results indicate that the distributions of independent variables are being compared, and a statistically significant *p-*value (less than 0.05) revealed the rejection of the null hypothesis for the variables, i.e., gender and nationality. The Mann-Whitney *U* test showed a significant difference (U = 110998.5, *p <* 0.05) between male and female students' interest in STEM. The scores for students' STEM interests differed in favor of the females, indicating that females showed greater interest in STEM than males. Similarly, the results yielded a significant difference (U = 57049.500, *p<* 0.05) between the national and expatriate student interest in STEM. The mean rank score was statistically significant for expatriate students than for nationals, indicating that expatriate students display more interest in STEM than their national counterparts do.

Furthermore, the study investigated student STEM interests across gender based on nationality. The results revealed no statistically significant difference in STEM interest among the Qatari male and female students (Table 6). However, a statistically significant difference was detected between expatriate male and female students, with expatriate female students more interested in STEM disciplines than males.

**Table 6:** Man- Whitney *U* test, investigating the STEM interest, gender, and nationality.

	Grou*p*	Sub-Grou*p*s	Test Statistics	Values
1.	National Students (n = 103)	Male	Mean rank	50.53
		Female	Mean rank	52.57
			Mann-Whitney U	1115.5
			*p*-value	0.755
2	Expatriate students (n = 970)	Male	Mean rank	561
		Female	Mean rank	409
			Mann-Whitney U	88957
		Male	*p*-value	<0.001*

**Note:** * Statistically significant; Sig. = significance level at 0.05.

### 3.3 Do the career aspirations of Qatar's high school students align with their STEM interests?

A STEM interest score (SIS) was calculated to investigate the career aspirations of students. The SIS was computed by totaling the survey's Likert coding responses on STEM interests. This included the subscales of the students' science, mathematics, and engineering & technology interests. The SIS scores ranged from −18 to +18, with a score of −18 indicating the least interest in STEM and +18 depicting the most interest in STEM. The maximum students' responses fell in the range of 7–12 (see Fig. 2). This SIS was also used for conducting a logistic regression to study the relationship between STEM interests and career aspirations.

Finally, bivariate logistic regression was conducted to see if students' STEM interests were correlated with their likelihood of pursuing a career in STEM fields [19]. In this analysis, the SIS score was the independent continuous variable, and career aspirations (STEM or non-STEM) were the dependent binary variable. Our results indicate that the increase in students' interest in mathematics and science is correlated with their likelihood to pursue a career in STEM fields: the higher the students' math and science interest are, the more likely they are to aspire to a STEM-related career. By contrast, student interest in engineering and technology doesn't seem to align with the likelihood of pursuing a STEM career (Table 7).

**Table 7:** Bivariate logistic regression illustrating the correlations between students' STEM interests and their likelihood to pursue a STEM career.

STEM interest	B	SE.	Wald χ2	Sig.	Exp(B)
Interest in Mathematics	.070	.020	12.491	<.001*	.932
Interest in Science	.138	.020	48.408	<.001*	.871
Interest in Engineering & Technology	.003	.016	.036	.849	.997
SIS (STEM interest score)	.062	.009	51.468	<.001*	0.940

**Note:** * Statistically significant; Sig. = significance level at 0.001.

**Abbreviations:** B relates to the coefficient for the model, Exp(B) relates to the odds ratio (i.e., probability of the event happening/probability of the event not happening).

## 4 Discussion

Our data analysis points to a context-dependent picture of the influences affecting student STEM interests and aspirations in Qatar. While our results reveal that students' nationality and gender were important predictors of interest in STEM fields of study and careers, no significant differences were observed for students' grade level, school type, and parental occupation. Overall, the results indicated that expatriate students were more inclined toward STEM than Qatari national students were. This contrast between the two student groups may signify two distinct patterns of career aspirations among the students.

Firstly, Qataris generally tend to prefer public (government) jobs or running a private business, than joining private sector employment [13]. Previous research has shown that Arab nationals generally have a penchant for the public over private sector jobs [12]. Preference for government jobs is ascribed to various reasons: high salaries, low expected productivity, job security, favorable work, etc. [40]. Indeed, the job market in the broader Arab region, including Qatar, has been criticized as displaying a “weak demand for skills and a strong demand for credentials” [41].

Secondly, expatriate students emerged more inclined toward STEM careers, compared to their Qatari counterparts. Different factors may account for this inclination. Qatar is home to many expatriates who make up a major segment of the country's population, with minimal prospects of permanent residency in the country. Students from expatriate households thus find themselves with few post-secondary educational choices inside Qatar, especially regarding STEM-related disciplines, due to the small number of higher education institutions in the country. Consequently, many leave Qatar to pursue higher education overseas, which requires rigorous academic performance, especially when planning to study STEM fields.

Available research demonstrates that non-Qatari students outperform Qataris, as is evidenced in a recent and past study by Ali et al. (2022), and El-Emadi et al. (2019) [42,43], causing a loss to the country's economic and social capital [44]. Similarly, student enrollment at Qatar University (QU), the only and most prominent national university in Qatar, tells a similar story. The number of students currently registered at QU stands at over 23,000 students: 66% of these are Qatari nationals, and 77% are females [11]. Looking closely at student data reveals that male students have continued to be attracted to Law, Mass Communication, and Engineering programs over the past decade. In contrast, females tend to opt for non-STEM programs (Primary and Secondary Education, Planning, and Development). Today, Law and Mass Communication remain the top academic options attracting students of both sexes.

The disconnect found in our study between students' aspirations to a career in STEM and their interest in engineering and technology may also be attributed to cultural influences which shape both fields are perceived in society. Arguably, society's attitudes and perceptions of engineering and technology disclose persistent gender stereotyping that reinforces the belief that women are not encouraged to work in these two fields. These stereotypes have been found to thwart women's entrance, participation, and persistence in STEM-related areas more generally [[45], [46], [47]].

Looking at gender-based differences regarding student interests and aspirations for all four STEM components combined, our analyses reveal that female students expressed more interest in STEM than males. In contrast, this result is not coherent with findings derived from the bulk of existing research, which has shown the male propensity to choose STEM-related subjects or careers [[48], [49], [50]]. Findings from available literature are inconclusive, for example, some studies depict that females tend to aspire to STEM careers more than males do [13,51]. Other research demonstrates female interest variations across the different STEM disciplines [52].

Our results revealed that while students' interest in mathematics and science is positively associated with their likelihood to aspire to a career in STEM fields, no association was detected between their interest in engineering and technology and their STEM-related career aspirations. These results also correspond with the study by Sorogo et al. (2018) [53]. Their study findings, based on Structural Equation Modeling, revealed that interest in Biology, Chemistry, and Physics can statistically significantly explain career aspirations as a researcher. However, Informatics, Mathematics, and Technology cannot [53]. Our study findings must be understood against the backlash of the current course offerings in Qatar's school system. Admittedly, the school curricula in public and private schools in the country don't incorporate engineering and technology subjects. The low interest in engineering and technology amongst students could also be due to the level of familial, specifically parental, support and motivation. As is well-documented in the literature, support, and encouragement from the family are important factors that foster children's STEM motivation [54,55]. Parents, particularly those in STEM occupations, impact their children's success and persistence in STEM fields [56]. Another factor that can explain females' low interest in the two subjects may be attributed to the lack of confidence in their abilities to perform well in both, i.e., their self-efficacy. Research demonstrates that high self-efficacy is a strong predictor of student interest, persistence, and success in STEM and has shown a self-efficacy gender gap often in favor of males [57].

## 5 Conclusion

The findings of the study indicated that 26% of high school students in Qatar aspire to be doctors, followed by a career in engineering (16%) and sports (13%). Our study's results further disclosed that the career the students aspire to the least is becoming a celebrity (0.1%). Interestingly, most females aspire to be doctors and males to be engineers. The disparity between the national and expatriate students has also seen revealed. Whereby most of the expatriate students aspire to be doctors while the same percentage of both Qatari and expatriate students aspire to be engineers. The second highest careers aspirations of Qatari students are to be a lawyer. furthermore, in general, females and expatriates showed greater interest in STEM than males and Qatari counterparts respectively. It is also noteworthy that no statistically significant difference in STEM interest among the Qatari male and female students was reported. However, a statistically significant difference was detected between expatriate male and female students, with expatriate female students being more interested in STEM disciplines than males. Finally logistic regression model depicted that the higher the students' math and science interests are, the more likely they are to aspire to a STEM-related career. By contrast, student interest in engineering and technology doesn't seem to align with the likelihood of pursuing a STEM career.

In light of the present study's findings, attention is called to attracting students, particularly the nationals to STEM-related subjects and professions. The results of this study and the recommendations that emerged from it are proposed to guide educational policymakers, curriculum developers, educators, and researchers in motivating and retaining students in STEM domains. In order to close the gender gap, there is a need to improve students' self-esteem and confidence in their abilities to do well (self-efficacy) in their STEM studies. This would involve acknowledging and praising their successes at school. To help them achieve their goals and cultivate their interest in STEM, project-based, interactive, and/or collaborative teaching methods can also aid in increasing student self-efficacy beliefs, compared to traditional approaches, such as lectures. Incorporation of inspiring academic and career role models in course teaching is an efficient tool to boost student confidence and spark their interest, inspiring students to take an active role in pursuing their dreams. Moreover, informal research experiences such as high school research experience programs (HSREP), undergraduate research experience programs (UREP), international conferences, science fairs, robotics contests, and STEM Olympiads must be encouraged because they are important predictors of STEM career choices [60]. Professional development programs for STEM teachers should also be provided to help them meet different pedagogical challenges. Last but not least, relevant career guidance and counseling must be provided to students to raise their awareness of potential STEM career choices.

The findings of this study need to be seen in light of some limitations. The first is methodological concerns i.e., exclusive reliance of the study on survey data. Wherein, using a qualitative or mixed-methods analysis would enrich the study. The study has focused on high school students' STEM interests and career pathways. However, no trajectories across the different academic levels have been mapped. To improve our understanding of students' motivational factors along with context-specific determinants of STEM interests and careers, further research is required. Furthermore, there is a need for longitudinal approaches to better understand possible variations in students' developmental trajectories to visualize alterations in students’ STEM interests and aspirations over time.

## Ethics committee name

Qatar University Institutional Review Board (QU-IRB).

## Approval code

QU-IRB 1424-EA/20.

## Author contribution statement

Abdellatif Sellami: Conceived and designed the experiments; Performed the experiments; Wrote the paper.

Malavika Santhosh; Zubair Ahmad: Analyzed and interpreted the data; Contributed reagents, materials, analysis tools or data; Wrote the paper.

Jolly Bhadra: Contributed reagents, materials, analysis tools or data; Wrote the paper.

## Funding statement

Open Access funding provided by the Qatar National Library

## Data availability statement

Data will be made available on request.

## Declaration of interest’s statement

The authors declare no conflict of interest.
