# Supplementary material for: High school students' STEM interests and career aspirations in Qatar: An exploratory study
Source: Heliyon. 2023 Feb 23;9(3):e13898. doi: 10.1016/j.heliyon.2023.e13898 (PMC10006460; doi:10.1016/j.heliyon.2023.e13898)
Supplement: Multimedia component 1 [file mmc1.pdf]

Supplementary materials

Fig S1: Students' questionnaire

PART I: STUDENT DEMOGRAPHICS

Q1. Gender

1. Male
2. Female

Q2. What is your date of birth?

(Day/ Month/ Year) ____ / ____ / ____

Q3. What is your nationality?

1. Qatari
2. Non-Qatari (Specify: ______________________)

Q4. What is your father/male guardian's highest education degree or certificate?

1. Never joined school
2. Elementary
3. Preparatory
4. Secondary
5. Post-secondary diploma
6. Bachelor's degree
7. Master's degree
8. Ph.D.

Q5. What is your father/male guardian's current employment status?

1. Full-time employee
2. Part-time employee
3. Unemployed, seeking a job **Go to → Q7**
4. Unemployed, not seeking a job **Go to → Q7**
5. Retired **Go to → Q7**
6. Unable to work **Go to → Q7**
7. Deceased
8. Business

Q6. What is your father/male guardian's main occupation?

1. STEM Field
2. Non-STEM Field ____

Q7. What is your mother's/female guardian highest education degree or certificate?

1. Never joined school
2. Elementary

3. Preparatory
4. Secondary
5. Post-secondary diploma
6. Bachelor’s degree
7. Master’s degree
8. Ph.D.

Q8. What is your mother’s/female guardian's current employment status?

1. Full-time employee
2. Part-time employee
3. Housewife **Go to → Q10**
4. Unemployed, seeking a job **Go to → Q10**
5. Unemployed, not seeking a job **Go to → Q10**
6. Retired **Go to → Q10**
7. Unable to work **Go to → Q10**
8. Deceased
9. Business

Q9. What is your mother’s/female guardian main occupation?

1. STEM Field
2. Non-STEM Field
3. Housewife

Q10. Which school type are you currently attending?

1. Government school / Independent school
2. Non-Government school / International / Private school

Q11. Which grade are you currently in?

1. Grade 11
2. Grade 12

Q12. Are you taking STEM courses?

1. Yes
2. No

Q13. Which of the following track are you taking?

[PROGRAMMER: multiple choice]

1. Biology
2. Chemistry
3. Mathematics
4. Physics
5. Engineering
6. Technology
7. Social science/humanities
8. General Science
9. Language

PART II: STEM INTEREST

PROGRAMMER: THIS SECTION IS MANDATORY

Q14. Please read the following statement related to mathematics and indicate to what extent you agree or disagree (Strongly Disagree; Disagree; Undecided; Agree; and Strongly Agree)

	Strongly Disagree	Disagree	Undecided	Agree	Strongly Agree
1. Maths has been my worst subject					
2. If I study hard, I will do well in maths					
3. I would consider a career that uses maths					
4. I know I can do well in maths					
5. I can handle most subjects well, but I cannot do a good job with maths					

Q15. In relation to maths, how would you rate the quality of the following? (Very poor; Poor; Fair; Good; Very good or Not Applicable)

	Very Poor	Poor	Fair	Good	Very Good
In-school events that are available (for example, summer schools and camps, lectures and debates, after-school clubs and maths clubs)					
Out-of-school events that are available (for example, trips and visits)					

Q16. Please read the following statement related to science and indicate to what extent you agree or disagree. (Strongly Disagree; Disagree; Undecided; Agree; and Strongly Agree)

	Strongly Disagree	Disagree	Undecided	Agree	Strongly Agree
Science has been my worst subject					
If I study hard, I will do well in science					
I would consider a career that uses science					
I know I can do well in science					
I can handle most subjects well, but I cannot do a good job with science					

Q17. In relation to science, how would you rate the quality of the following? (Very poor; Poor; Fair; Good; Very good or Not Applicable)

	Very poor	Poor	Fair	Good	Very good
In-school events that are available (for example, summer schools and camps, lectures and debates, after-school clubs and science clubs)					
Out-of-school events that are available (for example, trips and visits)					

Please read the following paragraph before you answer:

Engineers use math and science to invent things and solve problems. Engineers design and improve things like bridges, cars, machines, foods, and computer games.

Q18. Now, please read the following statements related to Engineering and indicate to what extent you agree or disagree. (Strongly Disagree; Disagree; Undecided; Agree; and Strongly Agree)

	Strongly Disagree	Disagree	Undecided	Agree	Strongly Agree
If I learn engineering, then I can improve things that people use every day					
I am interested in what makes machines work					
Designing products or structures will be important for my future work					
I am curious about how electronics work					
I would like to use creativity and innovation in my future work					

PART III: STEM BARRIERS

PROGRAMMER : THIS SECTION IS MANDATORY

Q19. Please read the following statements and indicate to what extent you agree or disagree. (Strongly Disagree; Disagree; Undecided Agree; and Strongly Agree)

	Strongly Disagree	Disagree	Undecided	Agree	Strongly Agree
**Society**					
Qatari society has a positive opinion on STEM pathway for male					
Qatari society has a positive opinion on STEM pathway for female					
Qatari society promotes success stories of male in STEM					
Qatari society promotes success stories of female in STEM					
**Family**					
My family encourages me to follow STEM pathway					
My family participates in activities involving STEM (magazines, television programs, visits to museums, etc.)					
In my family, we have conversations about what we are doing in school					
**Friends**					
1. My friends like STEM					

Q20. Please read the following statements and indicate to what extent you agree or disagree. (Strongly Disagree; Disagree; Undecided; Agree; and Strongly Agree)

	Strongly Disagree	Disagree	Undecided	Agree	Strongly Agree
1. **My teachers focus on a traditional approach to teaching the content**					
2. **My teachers use experiments in the classroom to explain the subject** (Teaching with experiments).					
3. **My teachers engage us through the investigation of the real-world challenges and problems** (Project-/Problem-based approach)					
4. **My teachers make us design and conduct our own scientific investigations**					

(Inquiry-Based Education)					
5. **My teachers involve us in teamwork / team projects**					
6. **My teachers provide us with opportunities to teach other students.** (Peer teaching)					
7. **My teachers use classroom time to discuss, challenge and apply ideas or knowledge from outside the class** (Flipped classroom)					
8. **My teachers tailor teaching to meet our individual interests and aspiration** (Personalized learning)					
9. **My teachers bring content and skills together** (Integrated learning)					
10. **My teachers use different activities in the classrooms** (Differentiated instruction)					

PART IV: FUTURE ASPIRATIONS

Q21. Which of the following do you consider your role model?

Please select up to three

1. Father	
2. Mother	
3. Brother	
4. Sister	
5. Uncle	
6. Aunt	
7. Male cousin	
8. Female cousin	
9. Male friend	
10. Female friend	
11. Male coach/teacher	
12. Female coach/teacher	
13. A famous scientist (male)	
14. A famous scientist (female)	
15. Other (please specify:________)	

Q22. What kind of work do you expect to be doing in the future? (Choose one answer only).

1. Join the Military
2. Join the Police
3. Accountant
4. Teacher
5. Lawyer
6. University Professor
7. Medical doctor

8. Nurse
9. Clerk
10. IT technician
11. Physical therapist
12. Chef
13. Scientist
14. Businessman/ Businesswoman
15. Diplomat
16. Engineer
17. Sportsperson
18. Celebrity
19. Other NON-STEM fields

Q23. How disruptive has the coronavirus outbreak been to your STEM learning?

1. Extremely disruptive
2. Very disruptive
3. Somewhat disruptive
4. Not disruptive at all

Q24. Please indicate the degree to which you agree or disagree that you are facing the following challenges in your STEM learning with the recent shift to remote/online learning.
(Strongly Disagree; Disagree; Undecided; Agree; and Strongly Agree)

**Statement**	Strongly Disagree	Disagree	Undecided	Agree	Strongly Agree
1. Keeping up with coursework					
2. Losing contact with teachers					
3. Communicating with staff/teachers					
4. Keeping a regular schedule while at home					
5. Being physically isolated from classmates					
6. Working on group projects					
7. Accessing & using technology					
8. Internet / IT technical problems					
9. Disruption at home					
10. Assessment styles used by teachers					

Do you have any questions or Comments?

Thank you ☺
